# Supplementary material for: The UK Biobank mental health enhancement 2022: Methods and results
Source: PLoS One. 2025 May 28;20(5):e0324189. doi: 10.1371/journal.pone.0324189 (PMC12118920; doi:10.1371/journal.pone.0324189)
Supplement: S1 File — (PDF) [file pone.0324189.s001.pdf]

Supplementary Material for:

“The 2022 Mental Health Enhancement for UK Biobank: Methods and Results” Davis, Coleman et al. 2025

Supplementary Figures: S1 Fig

Supplementary Tables: S1 – 6 Tables

Supplementary Figure 1

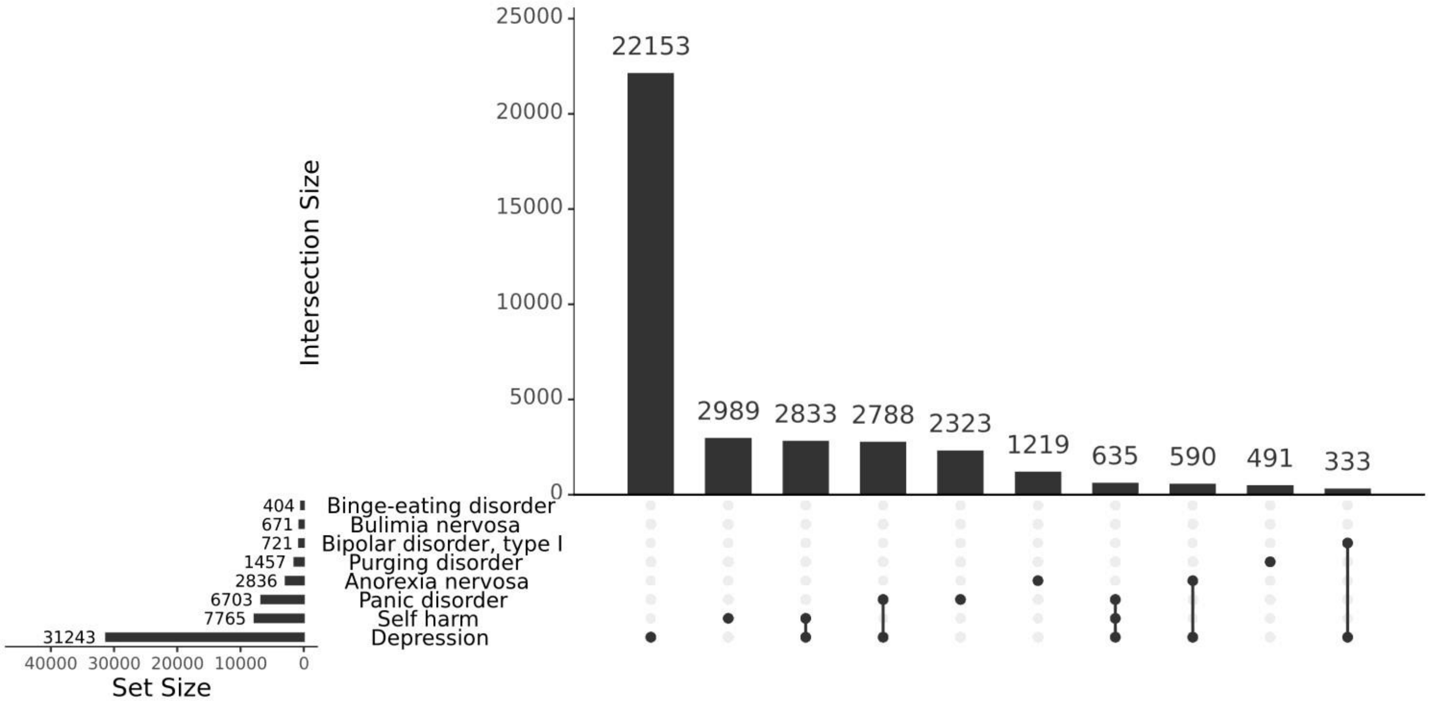

UPSET plots show the relative proportion and degree of intersection between concepts, in this case, phenotypes defined by symptom-based lifetime criteria in the MHQ2: depression, bipolar affective disorder type 1, panic disorder, and the four eating disorders, plus self-reported self-harm ever. The ten most common intersections are shown (out of a potential 127), which are mutually exclusive; for example, if a respondent met the criteria for lifetime depression, bipolar disorder and self-harm, they will not appear in the depression and self-harm or the depression and bipolar disorder intersects.

S1 Fig: An UPSET plot of lifetime phenotypes in MHQ2 showing the ten most common ‘combinations’.

S1 Table Respondents categorised by symptom-based criteria, which are not exclusive, with characteristics based on baseline questionnaire (BL) and second mental health questionnaire (MHQ2/Q2). Research team numbers n=165,253 ***Female only****:*

Characteristic	Overall	No lifetime criteria n (% have no criteria)	Depression n (% have depression)	Panic Disorder n (% have panic dx)	Any eating disorder n (% have eating dx)	Bipolar type I n (% have BP1 dx)
Female, n	97,646	71,372 (73%)	22,169 (23%)	4,913 (5.0%)	4,447 (4.6%)	473 (0.5%)
Personal characteristics						
**Age**[2]						
<65	27,915	17,680 (63%)	8,475 (30%)	2,201 (7.9%)	2,170 (7.8%)	237 (0.8%)
65-74	41,461	30,455 (73%)	9,307 (22%)	1,961 (4.7%)	1,797 (4.3%)	179 (0.4%)
>75	28,270	23,237 (82%)	4,387 (16%)	751 (2.7%)	480 (1.7%)	57 (0.2%)
Median	70	71	67	66	65	64
**Ethnicity**						
White	94,576	69,084 (73%)	21,510 (23%)	4,762 (5.0%)	4,296 (4.5%)	450 (0.5%)
Black	685	521 (76%)	141 (21%)	NA[1]	NA[1]	NA[1]
Asian	660	511 (77%)	122 (18%)	NA[1]	NA[1]	NA[1]
Chinese	261	218 (84%)	36 (14%)	NA[1]	NA[1]	NA[1]
Mixed	569	376 (66%)	163 (29%)	NA[1]	NA[1]	NA[1]
Other	543	414 (76%)	110 (20%)	151	151	23
Missing	352	248 (70%)	87 (25%)	NA[1]	NA[1]	NA[1]
[Combined][1]	NA	NA	NA	151 (4.9%)	151 (4.9%)	23 (0.1%)
Social factors measured at baseline						
Relative deprivation[3]	11,740	7,966 (68%)	3,199 (27%)	837 (7.1%)	766 (6.5%)	110 (0.9%)
Degree educated	41,807	29,803 (71%)	9,962 (24%)	2,054 (4.9%)	2,356 (5.6%)	216 (0.5%)
Rents home	4,540	2,759 (61%)	1,565 (34%)	448 (9.9%)	344 (7.6%)	73 (1.6%)
Longstanding illness	23,652	15,381 (65%)	7,272 (31%)	1,733 (7.3%)	1,239 (5.2%)	255 (1.1%)
Smoker	5,988	3,669 (61%)	1,989 (33%)	509 (8.5%)	425 (7.1%)	69 (1.2%)
Physical activity at least three times a week	34,611	25,070 (72%)	8,171 (24%)	1,807 (5.2%)	1,416 (4.1%)	172 (0.5%)
Neuroticism score, mean (sd)[4]	4.26 (3.16)	3.71 (2.92)	5.82 (3.29)	6.65 (3.28)	5.82 (3.32)	7.34 (3.37)
Social factors measured in MHQ2						
Childhood adverse experience[5]	41,575	26,404 (64%)	12,871 (31%)	3,162 (7.6%)	2,812 (6.8%)	360 (0.9%)
Adult adverse experience (lifetime)	27,742	16,214 (58%)	9,839 (35%)	2,480 (8.9%)	2,294 (8.3%)	292 (1.1%)
Social isolation	10,439	6,650 (64%)	3,314 (32%)	798 (7.6%)	638 (6.1%)	75 (0.7%)
Low resilience [6]	17,838	8,223 (46%)	8,586 (48%)	2,314 (13%)	1,497 (8.4%)	270 (1.5%)
Loneliness score, median (IQR) [7]	3 (3-5)	3 (3-4)	4 (3-6)	5 (3-6)	4 (3-6)	5 (3-7)
Self-rated health median (IQR) [8]	83 (72-91)	85 (75-92)	80 (64-89)	75 (60-86)	80 (66-90)	69 (45-82)

(1) To preserve participant privacy, non-White and missing ethnicities are collapsed to a single value ("Combined") if one cell size is smaller than 10.

(2) Age at MHQ2 is at date of completion of MHQ2

(3) Based on Townsend Deprivation Score of residence, where deprived □+2 (0 is average, and higher scores are more deprived)

(4) Any domain positive on the Childhood Trauma Screener

(5) Brief Resilience Scale, low = 1 to 2.99, where scale is 1-5

(6) Scores on at least two of three isolation questions

(7) Loneliness score from 3 to 9 points, with 3 being least lonely

(8) EQ-5D visual analogue scale, from 0 to 100, higher indicating better health

S2 Table: Respondents categorised by symptom-based criteria, which are not exclusive, with characteristics based on baseline questionnaire (BL) and second mental health questionnaire (MHQ2/Q2). Research team numbers n=165,253 ***Male only***

Characteristic	Overall	No lifetime criteria n (% have no criteria)	Depression n (% have depression)	Panic Disorder n (% have panic dx)	Any eating disorder n (% have eating dx)	Bipolar type I n (% have BP1 dx)
Male, n.	71,607	61,603 (86%)	9,074 (13%)	1,790 (2.5%)	315 (0.4%)	248 (0.3%)
Personal characteristics						
**Age[2]**						
<65	17,981	14,142 (79%)	3,457 (19%)	812 (4.5%)	167 (0.9%)	115 (0.6%)
65-74	29,001	24,988 (86%)	3,655 (13%)	681 (2.3%)	102 (0.4%)	85 (0.3%)
>75	24,625	22,473 (91%)	1,962 (8.0%)	297 (1.2%)	46 (0.2%)	48 (0.2%)
Median	71	72	67	66	64	66
**Ethnicity**						
White	69,219	59,521 (86%)	8,796 (13%)	1,731 (2.5%)	302 (0.4%)	232 (0.3%)
Black	438	400 (91%)	35 (8.0%)	NA[1]	NA[1]	NA[1]
Asian	788	721 (91%)	62 (7.9%)	NA[1]	NA[1]	NA[1]
Chinese	114	101 (89%)	13 (11%)	NA[1]	NA[1]	NA[1]
Mixed	294	227 (77%)	60 (20%)	NA[1]	NA[1]	NA[1]
Other	359	299 (83%)	54 (15%)	NA[1]	NA[1]	NA[1]
Missing	395	334 (85%)	54 (14%)	NA[1]	NA[1]	NA[1]
[Combined][1]	NA	NA	NA	59 (2.5%)	13 (0.5%)	16 (0.7%)
Social factors measured at baseline						
Relative deprivation[3]	8,275	6,693 (81%)	1,430 (17%)	319 (3.9%)	82 (1.0%)	55 (0.7%)
Degree educated	33,504	28,762 (86%)	4,318 (13%)	764 (2.3%)	166 (0.5%)	124 (0.4%)
Rents home	3,369	2,490 (74%)	800 (24%)	193 (5.7%)	50 (1.5%)	37 (1.1%)
Longstanding illness	20,644	16,720 (81%)	3,575 (17%)	780 (3.8%)	153 (0.7%)	139 (0.7%)
Smoker	5,873	4,727 (80%)	1,035 (18%)	242 (4.1%)	39 (0.7%)	47 (0.8%)
Physical activity at least three times a week	27,760	23,829 (86%)	3,570 (13%)	706 (2.5%)	122 (0.4%)	91 (0.3%)
Neuroticism score, mean (sd)[4]	3.36 (3.10)	3.01 (2.89)	5.55 (3.44)	6.49 (3.56)	6.46 (3.65)	6.46 (3.45)
Social factors measured in MHQ2						
Childhood adverse experience[5]	29,474	23,766 (81%)	5,196 (18%)	1,118 (3.8%)	207 (0.7%)	176 (0.6%)
Adult adverse experience (lifetime)	10,285	7,370 (72%)	2,626 (26%)	644 (6.3%)	131 (1.3%)	107 (1.0%)
Social isolation	6,926	5,449 (79%)	1,361 (20%)	279 (4.0%)	68 (1.0%)	44 (0.6%)
Low resilience [6]	8,529	4,880 (57%)	3,358 (39%)	813 (9.5%)	143 (1.7%)	136 (1.6%)

Loneliness score, median (IQR) [7]	3 (3-5)	3 (3-4)	4 (3-6)	5 (3-6)	5 (3-7)	5 (3-7)
Self-rated health median (IQR) [8]	84 (74-90)	85 (75-90)	79 (65-88)	74 (55-85)	73 (50-85)	71 (51-85)

(1) To preserve participant privacy, non-White and missing ethnicities are collapsed to a single value ("Combined") if one cell size is smaller than 10.

(2) Age at MHQ2 is at date of completion of MHQ2

(3) Based on Townsend Deprivation Score of residence, where deprived □ +2 (where 0 is average, and higher scores are more deprived)

(4) Any domain positive on the Childhood Trauma Screener

(5) Brief Resilience Scale, low = 1 to 2.99, where scale is 1-5

(6) Scores on at least two of three isolation questions

(7) Loneliness score from 3 to 9 points, with 3 being least lonely

(8) EQ-5D visual analogue scale, from 0 to 100, higher indicating better health

S3 Table: Age- and sex-stratified results for cases of depression, anxiety and harmful alcohol use in those who completed both questionnaires according to measures of current mental health (PHQ-9 and GAD-7 “last two weeks”, AUDIT “in the last year”)*

**Instrument**	**Depression (PHQ-9) case**				**Anxiety (GAD-7) case**				**Harmful alcohol use (AUDIT) case**			
**Sex**	**M**		**F**		**M**		**F**		**M**		**F**	
**Year**	**2016**	**2022**	**2016**	**2022**	**2016**	**2022**	**2016**	**2022**	**2016**	**2022**	**2016**	**2022**
**Overall N**	46,800	46,800	64,475	64,475	46,800	46,800	64,475	64,475	46,800	46,800	64,475	64,475
Positive	957	995	1670	1809	1400	1318	3118	3106	1891	1588	1155	968
Positive %	2.0%	2.1%	2.6%	2.8%	3.0%	2.8%	4.8%	4.8%	4.0%	3.4%	1.8%	1.5%
**Under age 52 N**	3,132	0	4,820	0	3,132	0	4,820	0	3,132	0	4,820	0
Positive	125	0	236	0	188	0	357	0	215	0	153	0
Positive %	4.0%	NA	4.9%	NA	6.0%	NA	7.4%	NA	6.9%	NA	3.2%	NA
**Age group 52-58 N**	8,358	4,048	13,660	6,184	8,358	4,048	13,660	6,184	8,358	4,048	13,660	6,184
Positive	299	186	579	346	407	256	912	529	482	252	369	203
Positive %	3.6%	4.6%	4.2%	5.6%	4.9%	6.3%	6.7%	8.6%	5.8%	6.2%	2.7%	3.3%
**Age group 59-65 N**	12,130	8,728	18,631	14,348	12,130	8,728	18,631	14,348	12,130	8,728	18,631	14,348
Positive	285	282	487	551	383	354	902	870	544	444	381	335
Positive %	2.3%	3.2%	2.6%	3.8%	3.2%	4.1%	4.8%	6.1%	4.5%	5.1%	2.0%	2.3%
**Age group 66-72 N**	16,459	12,614	20,450	19,034	16,459	12,614	20,450	19,034	16,459	12,614	20,450	19,034
Positive	177	240	306	450	322	325	764	817	520	473	220	278
Positive %	1.1%	1.9%	1.5%	2.4%	2.0%	2.6%	3.7%	4.3%	3.2%	3.7%	1.1%	1.5%
**Age group 73-79 N**	6,704	16,332	6,904	19,695	6,704	16,332	6,904	19,695	6,704	16,332	6,904	19,695
Positive	71	225	62	362	100	299	183	727	130	351	32	135
Positive %	1.1%	1.4%	0.9%	1.8%	1.5%	1.8%	2.7%	3.7%	1.9%	2.1%	0.5%	0.7%
**Age group >80 N**	17	5,076	10	5,207	17	5,076	10	5,207	17	5,076	10	5,207
Positive	0	62	0	100	0	84	0	163	0	68	0	17
Positive %	NA	1.2%	NA	1.9%	NA	1.7%	NA	3.1%	NA	1.3%	NA	0.3%

* Age at MHQ2 is at date of completion (NA where this was missing, n=28), age at MHQ1 is at approximate date of completing MHQ1

S4 Table: Relative risks of being a case on current mental health outcomes in MHQ2 ≈ 2022 compared to MHQ1 ≈ 2016 in age and sex specific groups in 2022 in people who completed both MHQs. Shading reflects size of effect. See ST3 for sample size by age and sex.*

	PHQ-9 Depression		GAD-7 Anxiety		Harmful alcohol use	
	M (n=46,800)	F (n=64,475)	M (n=46,800)	F (n=64,475)	M (n=46,800)	F (n=64,475)
Age* and sex specific **relative risk** positive result						
<52	NA	NA	NA	NA	NA	NA
52-58	1.28	1.33	1.29	1.28	1.07	1.22
59-65	1.39	1.46	1.28	1.27	1.13	1.15
66-72	1.73	1.60	1.30	1.16	1.16	1.36
73-79	1.27	2.00	1.20	1.37	1.11	1.40
>79	NA	NA	NA	NA	NA	NA
Sex specific **relative risk** positive result (including all age groups)						
All ages	1.05	1.08	0.93	1.00	0.85	0.83
Overall						
All ages and sex	1.07		0.98		0.84	

* *Age at MHQ2 is at date of completion (NA where this was missing, n=28), age at MHQ1 is at approximate date of completing MHQ1*

S5 and S6 Tables Comparison of lifetime conditions by participants, based on meeting criteria in MHQ1 and MHQ2 (restricted to those participants who answered both questionnaires)

*S5 Table: Comparison of lifetime conditions by participants, based on meeting criteria in MHQ1 and MHQ2, **Female only***

Case criteria	Met in both	Met 1 not 2	Met 2 not 1 *	Neither	Agree (%)	Kappa	New onset
**Depression**	10603	7850	4039	41983	82%	52%	1001 (25%)
**Bipolar**	102	255	176	63942	99%	32%	19 (11%)
**Self-harm**	2540	938	1266	59731	97%	68%	275 (22%)
**Cannabis**	11124	1995	1962	49394	94%	81%	162 (8.3%)
**Any SR clinician diagnosis**	17879	6877	2802	36917	85%	67%	NA

Met 1 = met criteria for lifetime phenotype in MHQ1, Met 2 = met criteria for lifetime phenotype in MHQ2, New = of those who met criteria in 2 and not 1, how many reported that their first experience was after the MHQ1 questionnaire or onset was unknown (was not asked with regards to clinician diagnosis)

*S6 Table Comparison of lifetime conditions by participants, based on meeting criteria in MHQ1 and MHQ2, **Male only***

Case criteria	Met in both	Met 1 not 2	Met 2 not 1 *	Neither	Agree (%)	Kappa	New onset
**Depression**	4009	3820	1908	37063	88%	51%	501 (26%)
**Bipolar**	55	193	103	46449	99%	27%	10 (10%)
**Self-harm**	958	473	522	44847	98%	65%	135 (26%)
**Cannabis**	10506	2020	1431	32843	93%	81%	92 (6.4%)
**Any SR clinician diagnosis**	7822	4801	1484	32693	87%	63%	NA

Met 1 = met criteria for lifetime phenotype in MHQ1, Met 2 = met criteria for lifetime phenotype in MHQ2, New = of those who met criteria in 2 and not 1, how many reported that their first experience was after the MHQ1 questionnaire or onset was unknown (was not asked with regards to clinician diagnosis)
